# From Lab to Industry: Advancing the Translational Potential of Extracellular Vesicles Through the ISEV Translation, Regulation and Advocacy Committee (ISEV‐TRA)

**DOI:** 10.1002/jev2.70220

**Published:** 2026-01-14

**Authors:** Uxia Gurriaran‐Rodriguez, Benedetta Bussolati, Mario Gimona, Konstantin Glebov, Yu Fujita, Antonio Marcilla, Christian Neri, Qing‐Ling Fu, Saumya Das, Stefano Pluchino, Natasa Zarovni, Carlos Salomon, Kenneth Witwer, Juan Manuel Falcon‐Perez

**Affiliations:** ^1^ Exosomes Laboratory Center for Cooperative Research in Biosciences (CIC bioGUNE) Basque Research and Technology Alliance (BRTA) Derio Spain; ^2^ Department of Medical Sciences University of Torino Torino Italy; ^3^ GMP Unit Paracelsus Medical University Salzburg Austria; ^4^ Research Program Nanovesicular Therapies Paracelsus Medical University Salzburg Austria; ^5^ Ludwig Boltzmann Institute for Nanovesicular Precision Medicine Salzburg Austria; ^6^ Exosome Consulting Bayreuth Germany; ^7^ School of Medicine Faculty of Health University of Plymouth Plymouth UK; ^8^ Division of Next‐Generation Drug Development Research Center for Medical Sciences The Jikei University School of Medicine Tokyo Japan; ^9^ Àrea de Parasitologia Departament de Farmàcia i Tecnologia Farmacèutica i Parasitologia. Facultat de Farmàcia i Ciències de l'Alimentació Universitat de València Valencia Spain; ^10^ Joint Research Unit on Endocrinology Nutrition and Clinical Dietetics Health Research IIS La Fe‐Universitat de València Valencia Spain; ^11^ Sorbonne Université, CNRS Inserm, Institut de Biologie Paris Seine (IBPS) Center for Neuroscience at Sorbonne Université (NeuroSU) Paris France; ^12^ Otorhinolaryngology Hospital of The First Affiliated Hospital Sun Yat‐sen University Guangzhou Guangdong China; ^13^ Extracellular Vesicle Research and Clinical Translational Center The First Affiliated Hospital Sun Yat‐sen University Guangzhou Guangdong China; ^14^ Center for Stem Cell Biology and Tissue Engineering Key Laboratory for Stem Cells and Tissue Engineering Ministry of Education Sun Yat‐Sen University Guangzhou Guangdong China; ^15^ Cardiovascular Research Center Mass General Hospital Boston Massachusetts USA; ^16^ Department of Clinical Neurosciences and National Institute for Health Research (NIHR) Biomedical Research Centre University of Cambridge Cambridge UK; ^17^ NEXUS project Day One srl Rome Italy; ^18^ RoseBio Srl Milan Italy; ^19^ Translational Extracellular Vesicles in Obstetrics and Gynae‐Oncology Group, The University of Queensland Centre for Clinical Research, Royal Brisbane and Women's Hospital, Faculty of Medicine The University of Queensland Brisbane Queensland Australia; ^20^ UQ Centre for Extracellular Vesicle Nanomedicine The University of Queensland Brisbane Australia; ^21^ Department of Molecular and Comparative Pathobiology Johns Hopkins University School of Medicine Baltimore Maryland USA; ^22^ Centro de Investigación Biomédica en Red de Enfermedades Hepáticas y Digestivas (Ciberehd) Madrid Spain; ^23^ Ikerbasque, Basque Foundation for Science Bilbao Spain

## Abstract

Extracellular vesicles (EVs) have emerged as powerful mediators of intercellular communication with significant potential across biomedical, veterinary, cosmetic, agricultural and environmental applications. However, the translation of EV‐based discoveries into practical and commercially viable products remains constrained by scientific complexity, regulatory uncertainty and manufacturing challenges. To address these barriers, the International Society for Extracellular Vesicles (ISEV) established the Translation, Regulation and Advocacy Committee (ISEV‐TRA). ISEV‐TRA aims to catalyse the responsible advancement of EV technologies by fostering cross‐sector collaboration, harmonising quality and regulatory frameworks and providing strategic advocacy to enhance market readiness. Through targeted initiatives—such as workshops and the development of translational resources and guidance—ISEV‐TRA seeks to bridge the gap between research and real‐world implementation. By promoting dialogue amongst academia, industry, investors and policymakers, ISEV‐TRA positions itself as a central driver in shaping the global roadmap for EV translation and commercialisation.

## Graphical abstract

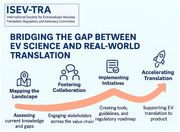

Extracellular vesicles (EVs) have emerged as critical mediators of biological processes, including cellular communication and have great potential as biomarkers and medicinal therapeutics for human health. EVs may also be used in veterinary settings, cosmeceuticals (skin regeneration and anti‐ageing), functional foods and nutraceuticals (EVs from plants, milk or probiotics), agricultural applications (to improve crop resistance or soil health), and environmental biotechnology for non‐agriculture applications (EVs as bio‐nanocarriers for bioremediation or bio‐sensing). Both academic spin‐outs and larger established companies are working towards bringing EV‐based products to market. However, despite this growing interest, significant challenges hinder the full‐scale translation of these discoveries into industry and society. To help address challenges in EV translation, the International Society for Extracellular Vesicles (ISEV) established the Translation, Regulation and Advocacy Committee **(ISEV‐TRA)** in 2024. The **ISEV‐TRA** aims to create an environment that facilitates evidence‐based and robust translation of EV research, complementing other ISEV initiatives such as the Regulatory Affairs and Clinical Use of EV‐based Therapeutics Task Force. **ISEV‐TRA** is composed of experts from academia, industry and consultancy, as well as individuals with regulatory and translational experience, ensuring a balanced representation of perspectives and expertise across the EV value chain. **ISEV‐TRA** seeks to identify key gaps and engage stakeholders across the entire value chain, laying the foundation for a comprehensive, unified innovation management, validation and regulatory framework, along with pursuing strategic advocacy.

A major obstacle in EV translational research is the reluctance of industry to invest in EV‐based technologies, largely due to unresolved regulatory and scientific gaps. Another obstacle can be limited knowledge on how EVs might be directly relevant for the mechanism at the heart of disease‐driving or disease‐resistance processes. To overcome this barrier, it will be essential to generate robust evidence that clearly demonstrates the competitive advantage of EV‐based products compared with existing market alternatives with similar applications. Academia faces persistent challenges, including the biological complexity of EVs, lack of standardised and fit‐for‐purpose separation and characterisation methods, and insufficient implementation of quality management systems (QMS), all of which contribute to reproducibility issues that are critical for translational approval. To strengthen credibility and accelerate progress, EV research and development must be conducted under robust QMS frameworks that embed documentation, traceability and quality controls from the outset. On the industry side, significant hurdles remain in scaling up manufacturing under good manufacturing practice (GMP) conditions and navigating regulatory uncertainty, as EV‐based therapies do not fit neatly into existing biologic or gene therapy categories, unlike more established products such as cell‐free DNA diagnostics, monoclonal antibodies or gene therapies. Limited understanding of EV pharmacokinetics and safety further complicates progress, underscoring the need for rigorous preclinical and clinical evaluation. In diagnostics, prognostics and monitoring, assays must be fit‐for‐purpose, meaning they can be reliably implemented in routine pathology laboratories. Rigorous analytical validation is required to ensure reproducibility and compliance with good laboratory practice (GLP) or Clinical & Laboratory Standards Institute (CLSI) guidelines. At the same time, regulatory bodies continue to face difficulties in defining clear safety and efficacy criteria for EV‐based technologies. Additionally, inconsistent regulations across countries hinder confidence, investment and commercialisation. While all sectors agree on the need for improved standardisation, quality control, and regulatory clarity, overcoming these challenges will require multidisciplinary collaboration and robust guidelines to advance EV technologies from research to practical application.

As a first step in mapping the current landscape of knowledge and interest in EV translation, **ISEV‐TRA** invited the EV community members, from a diverse cross‐section of researchers and industry professionals to complete a survey. Despite the size limitations, as respondents represented only around 5% of ISEV's >2000 members, the survey results have allowed **ISEV‐TRA** to highlight common challenges, emerging priorities and unmet needs in EV translation (10.1101/2025.10.31.685739). The survey revealed a strong cross‐sector demand for structured resources to support EV translational research, underscoring a potential role for **ISEV‐TRA** in leading and advocating for new initiatives. High‐priority needs include the development of a clear checklist for product translation and regular reports on the EV marketplace and commercialisation progress. Academia places particular emphasis on foundational guidance and consistent updates, while industry and dual‐affiliated respondents prioritise information on regulatory approvals, patents and clinical trial data, reflecting their focus on practical application and compliance. Notably, dual‐affiliated individuals express the strongest interest in expert insights on emerging EV opportunities, highlighting their unique position at the interface of research and commercialisation.

However, overall, the core challenge lies in understanding why EVs have yet to translate into commercial products, despite promising preclinical studies, numerous trials and substantial investment in the past 10 years. Most EV clinical trials to date have been small, investigator‐sponsored studies that never fully entered the mainstream regulatory pipeline of the FDA and EMA mainly. Consequently, even in 2025, EV therapies are viewed with scepticism by institutional investors and industry partners, with many companies struggling to secure funding or strategic partnerships. Thus, a central question remains: Are EV therapeutics still a viable investment in 2025? If not, why, and what evidence or innovation could change that? To address this, it is essential to examine honest, real‐world case studies that reveal what went wrong in translation and market viability. Addressing these questions through case studies and insights from opinion leaders will provide valuable guidance for both academic and industry stakeholders.

Therefore, **ISEV‐TRA** will leverage a focused symposium, ISEV × Pharma Connect Symposium, dedicated to addressing why EVs have struggled to reach the market despite substantial scientific and financial investment. The event will emphasise real‐world case studies that examine barriers to translation and market viability, aiming to provide actionable insights for both academia and industry. The symposium will feature four targeted sessions, each designed to genuinely bridge the gap between EV science and real‐world translation:

1. **From Mechanism to Product Definition**—This session will explore how to define an EV‐based product from the earliest stages by linking biological function to therapeutic potential. Discussions will focus on identifying the active component (API) and clarifying the mechanism of action (MoA) for EV products, to establish relevant potency assays and preclinical models that can predict clinical efficacy.
2. **Manufacturing and Analytical Strategies**—Addressing practical aspects of EV production and characterisation, this session will highlight scalable manufacturing approaches, quality control metrics, analytical tools and emerging technologies required for reproducibility and compliance. It will also serve as a platform for industry engagement and sponsorship, bringing forward perspectives from technology providers and manufacturers.
3. **Regulatory and Clinical Translation Lessons**—This session will showcase the real regulatory and clinical case studies, highlighting both successes and challenges encountered by EV‐based therapeutics. Comparative insights from other biologic modalities (e.g., cell and gene therapies) will be used to benchmark progress and outline regulatory expectations for safety, potency and consistency.
4. **Investment and Partnership Ecosystem Roundtable**—A candid, case‐driven dialogue focused on the investment landscape for EV technologies. This session will bring together venture investors, pharma innovation leads, CDMO executives, academic founders and regulatory strategists for an open discussion to examine the current scepticism amongst investors, discuss what evidence or innovation could restore confidence, and identify actionable steps to attract sustained partnerships between academia, biotech and pharma.

By emphasising real‐life case studies and bringing together key opinion leaders, policymakers, investors, CEOs and researchers, the symposium aims to foster open dialogue, identify critical bottlenecks and promote collaborative strategies that accelerate the translation of EV research into practical, commercially viable applications.

## Concluding Remarks

With access to a vast global network of key opinion leaders, **ISEV‐TRA** is uniquely positioned to bridge the gap between EV research and industry by proactively gathering and disseminating critical information to the research community to navigate the complexities of EV commercialisation more efficiently. In the longer term, this may include providing resources such as market reports, regulatory guidelines and commercialisation strategies to equip researchers and industry professionals with up‐to‐date, practical knowledge. Beyond knowledge sharing, the committee intends to contribute to the development of tools that can assist with legislation, regulation and advocacy, positioning **ISEV‐TRA** not only as a facilitator but also as a necessary driving force for translation. A key goal will be the establishment of a clear roadmap for EV product development across different application areas, ensuring that scientific rigour, regulatory requirements, and market feasibility are addressed in an integrated manner.

To further support translation, the **ISEV‐TRA** plans to consolidate information into a dedicated database or website and host workshops, such as the ISEV × Pharma Connect Symposium, to address key translational challenges and foster collaborative problem‐solving. As the field evolves, additional events may be organised to meet emerging needs. Through the creation of a centralised platform alongside workshops, networking events, and other knowledge resources, connecting researchers, industry leaders and stakeholders, **ISEV‐TRA** will enhance the visibility of EV‐based innovations, stimulate investment and accelerate their translation into impactful real‐world applications.

## Author Contributions

**Uxia Gurriaran‐Rodriguez**: writing – original draft, writing – review and editing, data curation, conceptualisation, formal analysis. **Benedetta Bussolati**: Writing – review and editing, conceptualisation. **Mario Gimona**: conceptualisation, writing – review and editing. **Konstantin Glebov**: conceptualisation, writing – review and editing. **Yu Fujita**: conceptualisation, writing – review and editing. **Antonio Marcilla**: conceptualisation, writing – review and editing. **Christian Neri**: conceptualisation, writing – review and editing. **Qing‐Ling Fu**: conceptualisation, writing – review and editing. **Saumya Das**: conceptualisation, writing – review and editing. **Stefano Pluchino**: conceptualisation, Writing – review and editing. **Natasa Zarovni**: conceptualisation, writing – review and editing. **Carlos Salomon**: conceptualisation; writing – review and editing. **Kenneth Witwer**: conceptualisation, writing – review and editing. **Juan Manuel Falcon‐Perez**: project administration, supervision, resources, conceptualisation, writing – original draft, writing – review and editing.

## Conflicts of Interest

The authors declare no conflicts of interest.

## Data Availability

The data that support the findings of this study are available from the corresponding author upon reasonable request.

